# Multiple tutorial-based assessments: a generalizability study

**DOI:** 10.1186/1472-6920-14-30

**Published:** 2014-02-15

**Authors:** Christina St-Onge, Eric Frenette, Daniel J Côté, André De Champlain

**Affiliations:** 1 Faculté de médecine et des sciences de la santé, Université de Sherbrooke, Sherbrooke, Canada; 2 Faculté des sciences de l’éducation, Université Laval, Québec, Canada; 3 Research and Development, Medical Council of Canada, Ottawa, Canada; 4 Chaire de recherche en pédagogie médicale de la Société des médecins de l’Université de Sherbrooke, Centre de pédagogie des sciences de la santé, Faculté de médecine et des sciences de la santé, Université de Sherbrooke, 3001, 12e Avenue Nord Sherbrooke, Sherbrooke, QC J1R 5 N4, Canada

**Keywords:** Assessment, G-study, Tutorial-based assessment, Programs of assessment

## Abstract

**Background:**

Tutorial-based assessment commonly used in problem-based learning (PBL) is thought to provide information about students which is different from that gathered with traditional assessment strategies such as multiple-choice questions or short-answer questions. Although multiple-observations within units in an undergraduate medical education curriculum foster more reliable scores, that evaluation design is not always practically feasible. Thus, this study investigated the overall reliability of a tutorial-based program of assessment, namely the Tutotest-Lite.

**Methods:**

More specifically, scores from multiple units were used to profile clinical domains for the first two years of a system-based PBL curriculum.

**Results:**

G-Study analysis revealed an acceptable level of generalizability, with g-coefficients of 0.84 and 0.83 for Years 1 and 2, respectively. Interestingly, D-Studies suggested that as few as five observations over one year would yield sufficiently reliable scores.

**Conclusions:**

Overall, the results from this study support the use of the Tutotest-Lite to judge clinical domains over different PBL units.

## Background

Tutorial-based assessment (TBA) is commonly used in small-group collaborative learning settings such as problem-based learning (PBL) curricula. PBL is a collaborative instructional format in which a group of students is brought together to achieve a common objective, for example, to solve a problem or to understand its underlying mechanisms [1,2].

TBA is thought to provide information about students that differs from that gathered with multiple-choice questions (MCQs), short answer questions (SAQs) or Objective Standardized Clinical Exams (OSCEs) for example [3-7]. More specifically, TBA has been used to measure clinical domains developed through PBL, such as interpersonal skills, reasoning, self-directed learning, communication and team interactions [1,4-6,8,9].

A literature search on TBA reveals several forms of varying length (e.g., 3 to 31 items) and proposed administration frequency (e.g. end of each session within a unit vs. at the end of a unit). Studies on the psychometric properties or qualities of the TBA are as varied as the methodologies used, which include for example inter-rater agreement, test-retest reliability and more comprehensive generalizability analyses. Comparing the reported coefficients can therefore be somewhat misleading, as they are measuring different facets of the TBA’s quality, appropriateness or purposefulness.

Notwithstanding the limited comparability of the observed coefficients, what can be gleaned from some of the studies on TBA is that more observations are better. More specifically, increasing the number of observations has been shown to improve the reliability of TBA [4,5]. Eva et al. [4] noted that assessment following each tutorial session (within a unit) resulted in decreased rater variability that was initially due to recall bias. They conducted a decision study (D-Study) which showed an increase in generalizability coefficient values when increasing the number of observations per unit. Similarly, Hebert and Bravo [5] showed, with Fleiss’ formula [10], that using a mean score based on at least five different unit assessments yielded an inter-rater agreement coefficient of 0.81, thus supporting the hypothesis that increasing the number of observations is preferable.

However, adopting the recommendation made by Eva and his collaborator [4] of having multiple observations within a unit may not always be feasible for a program. Therefore, following Hebert and Bravo’s [5] research, we propose a multiple-observation approach by considering the TBA completed at the end of different units as a program of assessment. That is, instead of considering the individual TBA when judging students’ PBL performance, all TBAs in a given period (for example a year or a specific phase within a program) should be aggregated. This approach to assessment is in-line with current preoccupations and developments that aim to promote the use of programs of assessment instead of individual assessment strategies [11-13]. This study investigated the overall generalizability of the Tutotest-Lite; a shortened version of the Tutotest [5] when considered as a program of assessment.

## Method

### Context

Tutorial-based assessment at the institution where this study was completed takes place during the first 30 months of a four-year system-based PBL curriculum that is composed of an 18-month clerkship (c.f. Table 1 for a list of the units – the different contents covered in the curriculum – per year, for the first two years). For each PBL unit (i.e., content), a new group is formed by randomly selecting 8 to 9 students (while maintaining a consistent male to female ratio).

**Table 1:** Tutotest-lite individual unit means and standard deviations for year 1 and year 2

**Year 1**			**Year 2**		
**Units**	**Mean**	***SD***	**Units**	**Mean**	***SD***
Introduction to the MD program	78.49	8.16	Cardiology	80.58	7.76
Basic science 1	79.97	9.18	Respiratory system	80.72	8.05
Basic science 2	82.14	9.96	Digestive system	78.81	6.78
Health and medicine for all age groups	69.30	11.73	Urology	78.61	6.51
Nervous system	80.26	8.24	Hematology	79.31	7.09
Psychiatry	79.07	7.50	Infectious diseases	78.73	6.17
Musculoskeletal system	80.03	7.69	Endocrinology	79.13	8.07
Public health	77.28	5.91	Ear nose and throat systems	80.20	7.45
Test grand mean	78.32	8.55	Test grand mean	79.51	7.24

Tutors are also randomly assigned to a random group of students and most often only serve as mentor for one unit. To ensure standardization of PBL units, all tutors are required to complete a two-day workshop on PBL principles and their application at the medical school where the study was conducted as well as a half-day of content specific training for each unit. Training is provided by the medical education center at this institution.

The Tutotest-Lite is one of four criteria (with overall average, success in immersion activities and attitude compatibility with the practice of medicine) used to determine promotion at the end of each of the first two years. Thus, a student is not promoted if the promotion committee convenes that he or she fails tutorial-based assessment (TBA). To make this decision, the promotion committee reviews students’ TBA scores for each unit of the academic year as well as tutor comments, and the performance pattern, using a qualitative global assessment perspective.

### The Tutotest-lite

The Tutotest-Lite was devised at the study institution to be a smaller, more manageable form of the Tutotest. The Tutotest, a 44-item tool completed at the end of a unit [5], was reviewed by members of the MD program evaluation committee. Clinical domains to be developed within PBL sessions were identified through focus groups composed of faculty members which were informed by the competency framework developed by the Royal College of Physicians and Surgeons of Canada, in order to have an overlap between clinical domains that should be focused on during PBL and those thought to be necessary for good medical practice.

**Figure 1:**
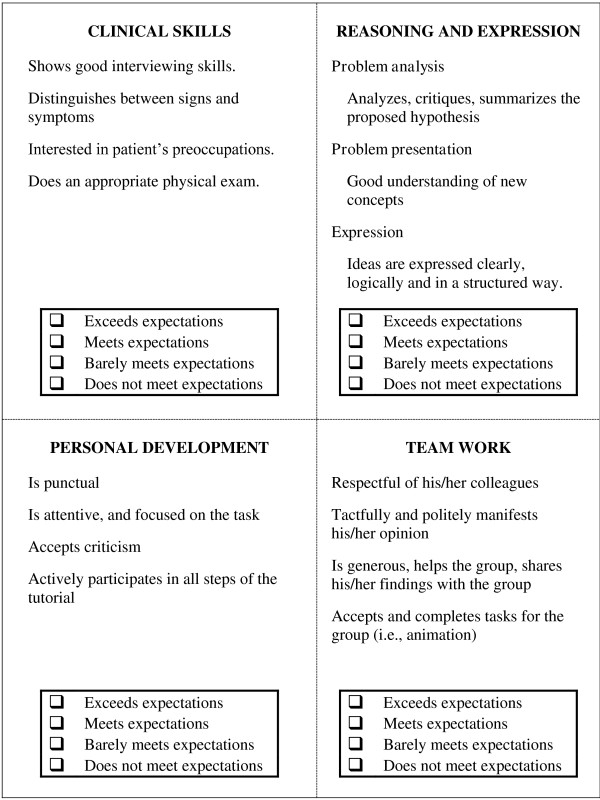
Extract from the Tutotest-Lite (clinical domains measured, scales used and examples of behavior descriptors).

The Tutotest-Lite is composed of four items that measure four clinical domains: clinical skills, reasoning and expression, personal development and team work (see Figure 1 for an extract of the Tutotest-Lite). Clinical skills are not taught and therefore not assessed in three of the eight units in Year 1 (i.e., the two basic science units and the introduction to the MD program unit). Four-point rating scales (1- does not meet expectations, 2- barely meets expectations, 3- meets expectations, and 4- exceeds expectations) are used to score these items. A list of score markers (i.e. behavioral descriptors for each score level) is provided on the form for the sole purpose of assisting tutors in completing the assessment. Tutors are free to use these descriptors as indicators of what they should observe during the PBL sessions. Tutors complete the evaluation for each student at the end of the unit. They are instructed to provide comments when they use the ratings 1 or 2; all tutors comply with this guideline. In addition, comments are provided for approximately 50% of the ratings.

### Data

Data for 399 first-year medical students and 385 second-year medical students were available. Two school years (2006–2007 and 2007–2008) were combined to increase the sample size for the analyses. Only complete data were used in this study. That is, when there were missing data for an entire unit, the student was eliminated from the data set. Data for 384 first-year and 374 second-year students were analyzed. Data were collected by the MD program where the study was conducted. De-identified raw data were provided to the principal investigator. Since this is a secondary analysis of anonymous data, the Canadian Tri-Council policy statement on ethical conduct for research involving humans state that consent is not required from participants [14].

### Analyses

Unit scores were computed for each student by adding item scores (each item measuring one ability). The units scores were transformed into percentages using the maximum score (i.e., 16). Descriptive statistics were calculated for unit percentage scores.

Univariate generalizability analyses were conducted [15] to assess the overall level of reliability and measure the relative importance of different sources of error. The following model was applied: students × units × clinical domains, where students is the object of measurement, and clinical domains and units are facets of generalization. Unit was treated as a fixed facet since all possible levels were exhausted [16]. Clinical domains, that is the score for each item on the form per unit, was treated as a random facet as elements were selected from a list of 44 items contained in the original Tutotest. Moreover, the generalizability of the observed performance was of interest. Since no information regarding tutors was gathered by the program, it was impossible to include this source of variability in the model. Analyses were conducted using G_String IV [17,18]. It is a user-friendly interface for urGenova [19] suitable for unbalanced designs. As previously noted, the form differed for three units of the first year. Thus, to have a balanced design, the analysis for Year 1 was conducted using a reduced form, that is using only the three common abilities in the eight units since systematic missing data cannot be legitimately incorporated in the model proposed. D-Studies were conducted to investigate g-coefficient values when varying the number observations (i.e. units).

## Results

Unit means in percentage metric and standard deviations are presented in Table 1. The average unit percentage scores mean was 78.91 with a 3.91 standard Deviation (SD) for Year 1 and 79.51 with a 0.86 SD for Year 2.

### Overall reliability

Generalizability coefficients, using the aforementioned evaluation design (students × units[fixed] × clinical domains[random]), were equal to 0.84 for Year 1 and 0.83 for Year 2. Variance components, which provide a measure of the relative importance of the different sources of variability (universe as well as error variances) are presented in Table 2. Students’ performance explained 10% and 8% of the variance respectively for Years 1 and 2 (universe score variance). Not surprisingly, the variance due to "case specificity" (i.e., the interaction between students and units [students × units]) explained most of the remaining score variability associated with the Tutotest-Lite scores for Year 1 and 2 (36% and 28% respectively). The other facets in the design explained little to none of the score variance.

**Table 2:** Tutotest-lite estimated variance component for years 1 and 2

		**Year 1**			Year 2*		
	**Variance components**	**df**	**Estimates**	**Percent of total variability**	**df**	**Estimates**	**Percent of total variability**
Students (s)	σ_s_	383	0.01950	10	373	0.01407	8
Units (u)	σ_u_	7	0.00294	2	7	0.00093	1
Clinical domains (c)	σ_c_	2	0	0	3	0.00630	4
s × u (case specificity)	σ_su_	2681	0.06787	36	2611	0.04591	28
s × c	σ_sc_	766	0.00403	2	1119	0.00512	3
c × u	σ_uc_	14	0.00014	0	21	0.00029	0
s × c × u + undifferentiated error variance	σ_scu, e_	5362	0.09295	50	7833	0.09305	56

*The analysis was conducted using only the same three clinical domains included in Year 1. We then observed a 0% variability for the clinical domain facet.

**Figure 2:**
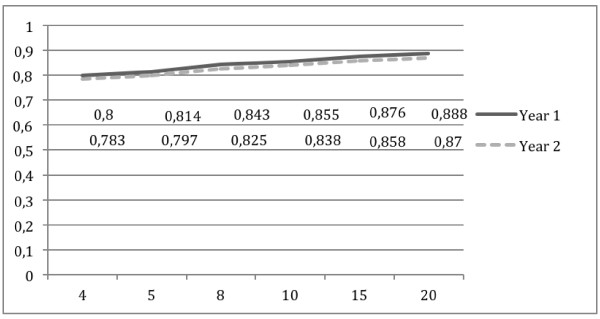
Decision-study results when varying the number of observations (i.e. units) for year 1 and year 2 Tutotest-Lite.

D-study results are presented in Figure 2. Tutorial-based assessment forms composed of three or four items appear to yield acceptable levels of reliability, that is, g-coefficients superior to 0.80. With respect to varying the number of observations, results in Table 2 show that a minimum of five observations is required to obtain a g-coefficient above 0.80. This was observed for both Years 1 and 2.

## Discussion

Tutorial-based assessment (TBA) is often used in PBL curricula to assess clinical domains not measured via traditional assessment tools. What can be gleaned from the literature is that more observations are better; however, multiple-observations within a unit are not always logistically feasible. The current study investigated the overall generalizability of the Tutotest-Lite when the observations from multiple units are used to judge clinical domains for a set period, specifically in the first two years of an organ-based PBL curriculum that promotes the development of complex skills, such as problem solving and team work.

The Tutotest-Lite showed overall good generalizability using our evaluation design (students × units[fixed] × clinical domains[random]); the g-coefficients exceeded 0.80, which is acceptable for the purpose of the present program. These values were observed in spite of low student variability because of the limited universe of generalization. That is, the objective was not to generalize the observed performance beyond the units included in the initial design as they are thought to be comprehensive and thus reflect the facet in its entirety.

The results obtained in this study add to the findings from Hebert and Bravo’s [5] observations in a number of ways. First, D-studies that were conducted showed that as few as five different TBA observations yield g-coefficients above 0.80 when using Tutotest-Lite (a 4-item form). These results, similarly to Hebert and Bravo [5], suggest that a minimum of five observations were required to achieve "acceptable" reliability. Interestingly, the same results as Hebert and Bravo [5] were achieved using a shorter form (4 items vs. the initial 44-item form), suggesting that it is more important to have multiple observations than longer forms.

Differences observed between Year 1 and 2 were small and it is thus difficult to speculate as to exactly which facet might account for the latter. Notwithstanding the limited generalisation of the following observations, observed g-coefficient differences might partly be due to the percentage of variance accounted for by student performances. Specifically, Year 2 showed a smaller percentage of variance explained by student performances than Year 1. Variance components show that student performances explained only 10% and 8% of the variance for Years 1 and 2 respectively. These results suggest that student performances have a somewhat lower score variability in the second year vs. the first year, though a restriction of range effect seems to be present in both cohorts. The percentage of variance due to case specificity (i.e., the interaction between students and units [students × units]) also varied between years 1 and 2 (36% vs. 28% for Year 1 and 2, respectively). A more detailed analysis of the topics covered in each year may shed some light on these results. However, it does appear that Year 1 units seem to cover more general, and therefore possibly more heterogeneous, topics (Introduction to the MD Program, Public Health, and Health and Medicine for all Age Groups, two units on Basic Science). However, topics covered in Year 2 are organ centered which may have contributed to more consistent performances across units, that is, a lesser impact of case specificity. Finally, the difference in percent variability for the facet clinical domain seems to be explained, at least in part, by the exclusion of one item in Year 1.

An important limitation of this study was the unavailability of tutor identifying information, that is, the MD program could not provide a dataset (to the principal investigator of this study) in which the PBL groups and their tutors were listed. It was therefore impossible to include this factor in the generalizability model and estimate its relative importance with regard to score variability. Moreover, the missing data created by trying to estimate the variance due to tutors (groups differed by tutor and tutor differed by unit, thus there was no consistency) could not have been treated in Generalizability theory. Similarly, information about group composition was not available to the researchers. Future research should collect data that would allow for the inclusion of this facet in the design. In addition, PBL membership, that is, group interaction, is another confounding factor that was not included in the analyses. PBL is a group activity for which it can be difficult to separate individual performances. A more cohesive group may produce better work that could yield higher scores from the tutor. This could contribute measurement error to the score variance that would not be accounted for in this study. Including group/rater as a factor might have highlighted rater stringency effects (i.e. did group performance vary systematically or randomly?). This effect may have been confounded with potential group dynamics, that is, a group performing better because of a greater degree of collegiality between individuals or higher competence in that given topic. More in-depth analysis, focused on using measures of group dynamics as covariates might shed some light on the importance of this source of measurement error in our design. Finally, generalization of these results should be done cautiously as the analysis was conducted at one medical school.

Finally, using the same items for each unit could yield an overestimate of the level of generalizability obtained in this study. Given that students are familiar with the items, they may have adapted their behavior to comply with the elements assessed. In other words, the measurement might have influenced the behavior of the students.

## Conclusion

The premise that "assessment drives learning" motivates test developers to devise purposeful assessment strategies. This was the underlying motivation of the present investigation which aimed to develop and assess some of the psychometric properties of the Tutotest-Lite. The results of this study replicated past findings with regard to the usefulness of including multiple observations per student. More specifically, our results support a program of assessment that incorporates multiple observations across PBL units when the universe of generalization is limited. Despite the limitations of this study, our results support the use of the Tutotest-Lite as a reliable tool to gather information in different settings to judge more appropriately the abilities and skills developed in PBL.

## Competing interests

The authors declare that they have no competing interests.

## Authors’ contributions

CSTO and DJC were responsible for data acquisition from the program. CSTO, EF and ADC were involved in the conception and design of the data analysis and interpretation of the results, and CSTO was responsible for the analysis. CSTO drafted the manuscript, all co-authors revised it critically and approved the final version.

## Authors’ information

CSTO is Chair holder of the Société des Médecins de l’Université de Sherbrooke Research.

Chair in Medical Education. DJC is coordinator of the assessment committee for the undergraduate medical education program at the Université de Sherbrooke. EF is an associate professor at Université Laval in assessment and measurement and is Editor of the journal *Mesure et évaluation en education*. ADC is the consulting chief research psychometrician at Medical Council of Canada.

## Pre-publication history

The pre-publication history for this paper can be accessed here:

http://www.biomedcentral.com/1472-6920/14/30/prepub
